# Modification of Hypoxic Respiratory Response by Protein Tyrosine Kinase in Brainstem Ventral Respiratory Neuron Group

**DOI:** 10.1371/journal.pone.0165895

**Published:** 2016-10-31

**Authors:** Hui Wang, Ruituo Huai, Junqing Yang, Yanchun Li

**Affiliations:** 1 Shandong Provincial Key Laboratory of Robotics and Intelligent Technology, Shandong University of Science and Technology, 579 Qianwangang Road, Qingdao, Shandong, P.R. China; 2 Department of Neurobiology & Anatomy, Drexel University College of Medicine, Philadelphia, PA 19129, United States of America; Rutgers University, UNITED STATES

## Abstract

Protein tyrosine kinase (PTK) mediated the tyrosine phosphorylation modification of neuronal receptors and ion channels. Whether such modification resulted in changes of physiological functions was not sufficiently studied. In this study we examined whether the hypoxic respiratory response—which is the enhancement of breathing in hypoxic environment could be affected by the inhibition of PTK at brainstem ventral respiratory neuron column (VRC). Experiments were performed on urethane anesthetized adult rabbits. Phrenic nerve discharge was recorded as the central respiratory motor output. Hypoxic respiratory response was produced by ventilating the rabbit with 10% O2-balance 90% N2 for 5 minutes. The responses of phrenic nerve discharge to hypoxia were observed before and after microinjecting PTK inhibitor genistein, AMPA receptor antagonist CNQX, or inactive PTK inhibitor analogue daidzein at the region of ambiguus nucleus (NA) at levels 0–2 mm rostral to obex where the inspiratory subgroup of VRC were recorded. Results were as follows: 1. the hypoxic respiratory response was significantly attenuated after microinjection of genistein and/or CNQX, and no additive effect (i.e., further attenuation of hypoxic respiratory response) was observed when genistein and CNQX were microinjected one after another at the same injection site. Microinjection of daidzein had no effect on hypoxic respiratory response. 2. Fluorescent immunostaining showed that hypoxia significantly increased the number of phosphotyrosine immunopositive neurons in areas surrounding NA and most of these neurons were also immunopositive to glutamate AMPA receptor subunit GluR1. These results suggested that PTK played an important role in regulating the hypoxic respiratory response, possibly through the tyrosine phosphorylation modification of glutamate AMPA receptors on the respiratory neurons of ventral respiratory neuron column.

## Introduction

Protein tyrosine kinases (PTKs) are important enzymes that integrally participated in the regulation of cell proliferation, cell growth, cell cycle, immune responses and a variety of intracellular signaling mechanisms [1]. PTK mediates the enzymatic transfer of the γ phosphate of ATP to the phenolic groups on tyrosine residues to generate phosphate monoesters. It is expressed within the CNS and associated with synapses, suggesting roles in neuronal function [2, 3]. Protein tyrosine phosphorylation is a key biochemical event in several cellular processes including proliferation, growth, and differentiation [4]. Studies showed that tyrosine kinase receptor B agonist pretreatment enhanced neuronal survival and long-term sensory motor function following hypoxic ischemic injury in neonatal rats [5]. Inhibition of Src kinase attenuated the neuroinflammatory response after hypoxic injury [6]. However, the role of PTK in modulating hypoxic chemoreflex has not been studied.

Our previous studies have shown that inhibition of PTK at brainstem solitary tract nucleus caused significant attenuation of hypoxic respiratory response [7]. Although solitary tract nucleus is the major relay station of the peripheral chemoreceptors, peripheral chemoafferents were also observed to directly project to the ventral respiratory neuron column (VRC) that is anatomically associated with the ambiguus nucleus (NA) [8]. Since neural signal transmission along the central pathway of peripheral chemoreflex that mediated the hypoxic respiratory response was mainly mediated by glutamate AMPA receptors [9–11], we hypothesized that at the VRC/NA, PTK might regulate the hypoxic respiratory response by mediating the tyrosine phosphorylation of AMPA receptors. In this study we will observe whether inhibition of PTK at VRC/NA attenuates the hypoxic respiratory response and whether blockade of AMPA receptor alternates the effect of PTK inhibition, and whether PTK and AMPA receptors are co-expressed in neurons in VRC/NA.

## Materials and Methods

### General procedures

Experiments were performed on adult rabbits (New Zealand white, weighing 2.2–2.6 kg, n = 36) of either sex. Animals were bred in Laboratory animal center with free access to food and water. The rabbit was anesthetized with urethane at initial dose of 1.0 g/kg (i.v.). A supplemental dose (0.1 g/kg, i.v.) was given whenever the rabbit showed responses to clamp at the hind limb or noxious stimuli. The use of urethane and all procedures conformed to the Guidelines for the Use of Animals of the International Brain Research Organization and were approved by the Institutional Animal Care and Use Committee of Shandong University of Science and Technology (No.201302). Trachea was cannulated to facilitate ventilation. The femoral artery and vein were cannulated for monitoring arterial pressure, withdrawing blood for blood gases and for drug administration. The animal’s head was fixed on a stereotaxic apparatus (Narishige SN-3, Japan). The dorsal surface of the medulla was exposed by occipital craniotomy. Dura and arachnoid membranes were gently removed. The exposed brain was covered with warmed paraffin oil (37–38°C) to avoid drying. Mean arterial blood pressure was monitored and maintained at 100 ± 10 mmHg by intravenous infusion of 0.9% saline. The animal breathed spontaneously.

### Phrenic nerve recording and hypoxia test

The C5 branch of the phrenic nerve were isolated and severed through a dorsal approach. The central end of phrenic nerve was mounted onto bipolar silver recording electrode immersed in mineral oil to avoid drying. Phrenic discharge was amplified, filtered (50–3000Hz) and displayed on a dual-bean memory oscilloscope (VC-10, Nihon Kohden). Phrenic signals were then sampled into a computer for recording and analyzing with Biobench software (NI Corporation USA).

Hypoxia test was applied by switching the ventilation gas from room air to 10% O_2_-balance 90% N2 for 5 minutes. At the end of the 5-minutes hypoxia, the arterial O_2_ partial pressure was reduced from 104 ± 13 mmHg to 43 ± 6 mmHg (n = 5, P < 0.01).

### Microinjection

All microinjections of drugs were accomplished with glass micropipettes (tip outside diameter 20–30 μm) guided by stereotaxic coordinates. To identify the optimal site for microinjection, a monopolar tungsten electrode was inserted into NA (0–2.0 mm rostral to the obex, 3.0–4.0 mm lateral and at 3.0–5.0 mm below the dorsal surface) to record multiunit inspiratory discharges. Microinjection was made at site where inspiratory discharges were recorded. The microinjection pipette was connected to a PPM-2 pneumatic pressure injection pump module (Neurophore BH-2, Harvard Apparatus, Holliston, MA, USA) for pressure injection. The injection volume was measured by observing meniscus movements within the pipette under an operation microscope equipped with graticule in the eyepiece. All drugs were purchased from Sigma (St Louis, MO, USA). CNQX was dissolved in artificial cerebrospinal fluid (ACSF) at concentration of 10 mM. Genistein or daidzein was dissolved in dimethyl sulfoxide (DMSO) and then diluted in ACSF to final concentration of 0.5 mM (DMSO <5%). The injection volume for each injection was 100 nl.

### Histological verification of injection sites

**Fig 1:**
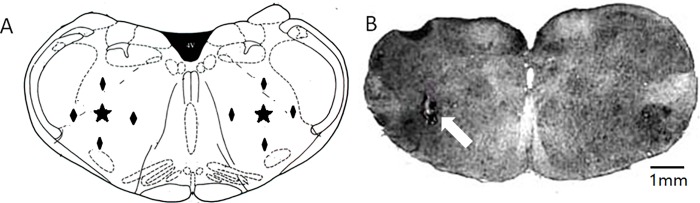
Locations of microinjection sites. A: Schematic drawing at a level of 2 mm rostral to the obex. Microinjection sites were indicated with ★. Control injection sites were indicated by ◆. B: A representative photomicrograph showing the actual injection site marked with a lesion (arrow).

At the end of each experiment, a D.C. current (20 μA for 30 sec) was passed through the injection pipette to produce an electrolytic lesion marking the injection sites. The animals were killed with urethane overdose (2 g/kg, i.v.) and transcardially perfused with normal saline followed by 10% formalin solution. The brain was removed, post-fixed in formalin and cut into 40-μm frozen sections. The locations of injection sites were identified under bright-field microscope (Fig 1).

### Data analysis

The inspiratory amplitude (represented by the amplitude of integrated phrenic nerve discharge, ∫Phr) and respiratory frequency (number of respiratory cycles in 1 minute) were measured and averaged every minute. Changes in ∫Phr and respiratory frequency (*f*) in response to hypoxia were expressed as percentages of changes over pre-hypoxia baseline values. Data was expressed as means ± SD. Student t-test and F-test were used to decide the statistic significance, which was set at P < 0.05.

### Fluorescence immunostaining experiment

Animals were divided into four groups randomly: the normal ventilation group and hypoxia groups 1–3 (10% O_2_-balance 90% N_2_ ventilation for 5, 30, 60 minutes, respectively). Then all animals were killed with urethane overdose (2 g/kg, i.v.) and the brainstems were quickly removed and cut into 20-μm coronal sections on a freezing microtome.

Five consecutive sections that were cut from 1 to 1.1 mm rostral from obex were immunostained. Briefly, the sections were fixed in cold acetone, rinsed, and blocked in 10% normal goat serum in containing 0.2% Triton X-100. For single staining experiment, the sections were incubated in mouse anti-phosphorylated tyrosine antibody (diluted at 1:100, Santa Cruz, USA) overnight at 4° C, rinsed, and then in FITC-conjugated goat anti-mouse IgG (1:100, Santa Cruz, USA) for 40 minutes at room temperature.

For double staining experiment, the sections were incubated in a mixture of mouse anti- phosphorylated tyrosine antibody (1:100) and rabbit anti-GluR1 antibody (1:100, Santa Cruz, USA) overnight at 4° C, rinsed, and then in a mixture of FITC-conjugated goat anti-mouse IgG (1:100, Santa Cruz, USA) and Cy3 conjugated goat anti-rabbit IgG (1:100, Santa Cruz, USA) for 40 minutes at room temperature.

The fluorescent images were captured under a confocal microscopy (Olymus, Japan). The number of immunopositive neurons within a 1-mm diameter circle around the NA were counted by double blind method and was expressed as the mean ± SD. The data were analyzed by independent-sample t test of SPSS 11.0 software.

## Results

### Microinjection of PTK inhibitor genistein attenuated hypoxic respiratory response

**Fig 2:**
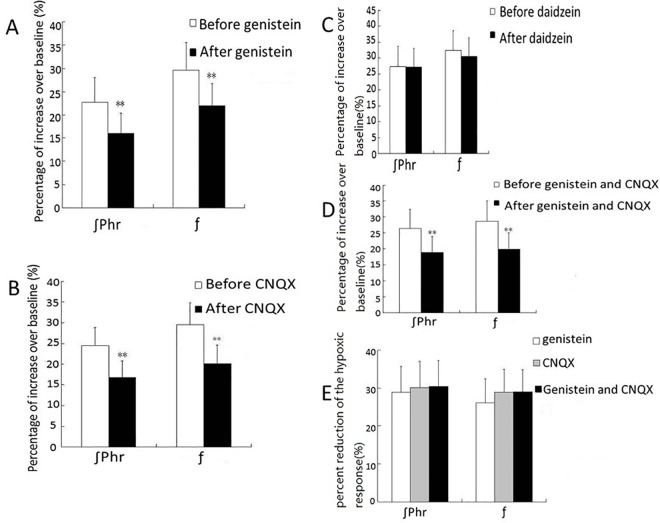
Effects of microinjection of different drugs into VRC/NA on hypoxic respiratory response. A-C: Hypoxic respiratory response (the increases of ∫Phr and *f* during hypoxia test) was decreased after microinjection of genistein (A, n = 10) or CNQX (B, n = 10), but not after daidzein (C, n = 6). D-E: CNQX and/or genistein, when microinjected together or one after another, did not cause stronger suppression of hypoxic respiratory response than when microinjected alone.

All rabbits were given a standard hypoxia test (breathing 10% O_2_-balance 90% N_2_ for 5 minutes) under control condition for their normal hypoxic respiratory response. During the hypoxia test, the average ∫Phr and average *f* were increased compared with those under normoxia condition (Table 1, n = 36). After the animal’s breathing (phrenic discharge) recovered completely back to pre-hypoxic level (usually 15 minutes after the hypoxia test), PTK inhibitor genistein (0.1 μl, 0.5 mM) was microinjected into VRC/NA. 5 minutes after the microinjection, another hypoxia test was performed. During this hypoxia test, both ∫Phr and *f* were still increased but the increases were significantly smaller than the control hypoxic respiratory response. On average, the increase of ∫Phr was decreased by 28.91 ± 6.79% (n = 10, P < 0.01) and the increase of *f* was decreased by 26.09 ± 6.33% (n = 10, P < 0.01) (Table 1, Fig 2A).

**Table 1:** Effects of microinjections of genistein and CNQX at VRC/NA on hypoxic respiratory response.

Hypoxia Response					
	n	Before microinjection (Control)		After microinjection	
		∫Phr	*f*	∫Phr	*f*
Genistein alone	10	22.74 ± 5.31%	29.66 ± 5.95%	16.02 ± 4.38%**	21.97 ± 4.73%**
CNQX alone	10	24.52 ± 4.29%	29.58 ± 5.23%	16.82 ± 4.04%**	20.14 ± 4.47%**
Genistein + CNQX	10	26.46 ± 5.99%	28.74 ± 6.39%	18.95 ± 5.01%**	19.93 ± 5.11%**
Daidzein	6	27.35 ± 7.22%	27.19 ± 6.87%	32.41 ± 11.02%	30.54 ± 10.57%

All values are percentage of changes over pre-hypoxia baseline.

**P < 0.01 vs. Control. P > 0.1 between genistein+CNQX and genistein alone, and between genistein + CNQX and CNQX alone.

### Microinjection of PTK inactive inhibitor daidzein had no effect on hypoxic respiratory response

After microinjection of daidzein (0.1μl, 0.5 mM) at VRC/NA, hypoxia still caused increases of ∫Phr and *f* and the increases were not significantly different from the control hypoxic respiratory response (n = 6, P > 0.05) (Table 1, Fig 2C).

### Microinjection of CNQX attenuated hypoxic respiratory response

After microinjection of glutamate AMPA receptor antagonist CNQX (0.1 μl, 10 mM) at VRC/NA, hypoxia test caused smaller hypoxic respiratory response. The increases of ∫Phr and *f* were decreased respectively by 30.11 ± 6.95% (n = 10, P < 0.01) and 28.91 ± 6.07% (n = 10, P < 0.01) (Table 1, Fig 2B).

### Microinjection of CNQX following the microinjection of genistein at the same injection site did not further attenuate the hypoxia respiratory response

In another group of animals, microinjection of CNQX (0.1μl, 10 mM) was performed 2 minutes after the microinjection of genistein (0.1μl, 0.5 mM) at the same injection site at VRC/NA. 5 minutes after such microinjections of genistein and CNQX, hypoxia test still caused hypoxic respiratory response, i.e., the increases in ∫Phr and *f*. Compared with control hypoxic respiratory response, the increase of ∫Phr was decreased by 30.44 ± 6.82% (n = 10, P < 0.01) and the increase of *f* was decreased by 29.01 ± 5.84% (n = 10, P < 0.01), only slightly stronger than when genistein was microinjected alone and the differences were not statistically significant (P > 0.1) (Table 1, Fig 2D and 2E).

### Control experiments

Respiratory parameters (∫phr and f) were not significantly changed after microinjections of ACSF or ACSF containing 5% DMSO at VRC (∫phr 28.69 ± 5.97% vs 28.32 ± 6.22%, f 31.41 ± 6.12% vs 30.97 ± 5.46%, n = 5, P > 0.1), or microinjections of genistein/CNQX at loci 1.5 mm away from the VRC (for genistein, ∫phr 27.86 ± 7.07% vs 30.32 ± 8.23%, f 32.69 ± 0.06% vs 30.04 ± 0.54%, n = 5, P > 0.1; for CNQX, ∫phr 29.43 ± 3.23% vs 31.29 ± 6.33%, f 31.18 ± 3.69% vs 29.09 ± 6.33%, n = 5, P > 0.1).

### Expression of phosphotyrosine immunopositive neurons in VRC/NA

**Fig 3:**
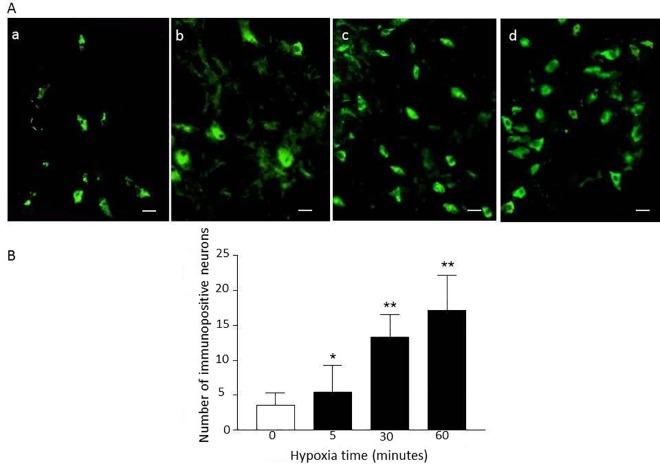
Phosphotyrosine immunopositive neurons in VRC/NA. A: Photomicrographs showing the phosphotyrosine immunopositive neurons (FITC fluorescent) in area of VRC/NA. a-d: hypoxia times of 0 (control), 5, 30, and 60 minutes, respectively. Scale bar, 20μm. B: The number of phosphotyrosine immunopositive neurons increased with the increase of hypoxia challenge times in hypoxia groups (** P< 0.01 and * P<0.05, vs. control group. Each group n = 5).

Phosphotyrosine immunopositive neurons were observed in all animals in area corresponding to the inspiratory subgroup of VRC/NA. The diameters of immunopositive neurons were 15–20 μm and they were mostly polygonal (Fig 3A). The number of immunopositive neurons in the VRC/NA area were only 3.58 ± 1.77/section in normal ventilation group but increased significantly in hypoxia-tested groups (5.43 ± 2.83 in 5 minutes hypoxia group, 13.37 ± 2.83 in 30 minutes hypoxia group, and 17.14 ± 5.07 in 60 minutes hypoxia group) (Fig 3B).

**Fig 4:**
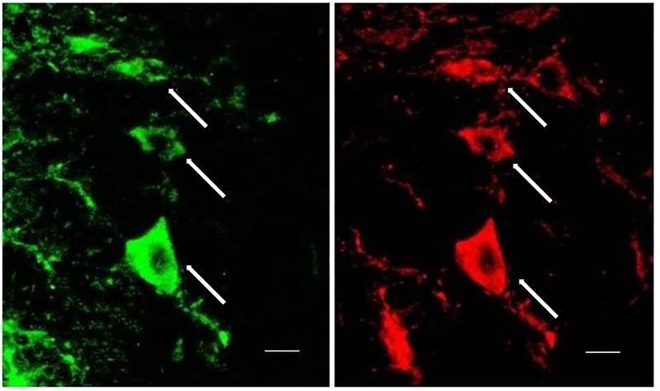
Phosphotyrosine and GluR1 immunopositive neurons in VRC/NA. Photomicrographs showing that the phosphotyrosine immunopositive neurons (FITC, green) were also immunopositive to GluR1 (cy3, red). Arrows indicated the double-labeled neurons. Scale bar, 10μm.

Almost all phosphotyrosine immunopositive neurons were also immunopositive to GluR1, i.e. they were double-labeled for both phosphotyrosine and GluR1 (Fig 4). The number of such double-labeled neurons in VRC/NA increased with the increase of hypoxia times in hypoxia groups in an identical pattern as phosphotyrosine single-labeled neurons.

## Discussion

This study demonstrated that microinjection of PTK inhibitor genistein at VRC/NA attenuated the hypoxic respiratory response and this effect was not influenced by further blockade of glutamate AMPA receptor with CNQX at the same microinjection site. We suggested that the effect of PTK blockade on hypoxic respiratory response could be due to the de-phosphorylation and inactivation of AMPA receptors.

Receptor phosphorylation is a common mechanism for regulating the receptor function and synaptic transmission. Accumulating evidence indicated that the AMPA receptor function could be regulated by direct phosphorylation by serine/threonine kinases [12], protein kinase C (PKC), protein kinase A (PKA), and especially the PTK [13–17]. Tyrosine phosphorylation modification of AMPA receptor subunits enhanced the AMPA receptor mediated synaptic transmission by increasing the AMPA receptor opening time and the AMPA receptor mediated current, and by promoting the membrane insertion and sequestration of the receptors to postsynaptic membrane. On contrary, de-phosphorylation of AMPA receptor subunits weakened the synaptic transmission. The degree of tyrosine phosphorylation of AMPA receptor subunits was balanced by the activity of PTK that promoted phosphorylation, and the activity of phosphatase that promoted the dephosphorylation. Inhibition of PTK will move the balance toward dephosphorylation, resulting in dephosphorylation of the AMPA receptor subunits and weakening of synaptic transmission.

Glutamate AMPA receptor mediated the fast synaptic transmission in the central nervous system [18]. It played a critical role in maintaining the excitability of the brainstem respiratory neuronal networks of VRC that generated respiratory rhythm and drive the inspiratory and expiratory activity [9, 10, 19]. Blockade of AMPA receptors at VRC caused severe depression of inspiration, prolongation of expiration, and even complete apnea, depending on the VRC subgroups being blocked [20]. In this study we only blocked the AMPA receptors at the inspiratory subgroup of VRC (in vicinity of NA at levels 0–2 mm rostral to obex) by microinjecting CNQX, resulting in moderate suppression of inspiration and attenuation of hypoxic respiratory response. However, after microinjection of PTK inhibitor genistein at VRC/NA inspiratory subgroup that caused attenuation of hypoxic respiratory response, subsequent microinjection of CNQX at the same injection site did not cause further attenuation of the hypoxic respiratory response. This could be due to the near-complete deactivation of AMPA receptors after the inhibition of PTK. Since the AMPA receptors at the injected region were already deactivated, subsequent blockade of the receptors at the same region would not produce additional attenuation of hypoxic respiratory response.

Phosphotyrosine immunopositive neurons were observed in area corresponding to the inspiratory subgroup of VRC/NA, and the number of such neurons increased with the increasing times of hypoxia challenge. In addition, most phosphotyrosine immunopositive neurons were also immunopositive to AMPA receptor subunit GluR1. These findings provided further support that the PTK mediated tyrosine phosphorylation modification of AMPA receptor subunit was involved in the regulation of hypoxic respiratory response.

## Conclusion

This study demonstrated that the PTK mediated phosphotyrosine modification is associated with the regulation of hypoxic respiratory response in vivo, probably by modifying the functions of glutamate AMPA receptors in neurons of VRC/NA.
